# HIV-Associated Disseminated Histoplasmosis and Rare Adrenal Involvement: Evidence of Absence or Absence of Evidence

**DOI:** 10.3389/fcimb.2021.619459

**Published:** 2021-03-15

**Authors:** Mathieu Nacher, Kinan Drak Alsibai, Audrey Valdes, Philippe Abboud, Antoine Adenis, Romain Blaizot, Denis Blanchet, Magalie Demar, Félix Djossou, Loïc Epelboin, Caroline Misslin, Balthazar Ntab, Nadia Sabbah, Pierre Couppié

**Affiliations:** ^1^Centre d’Investigation Clinique (CIC) INSERM 1424, Centre hospitalier Andree Rosemon Cayenne, Cayenne, French Guiana; ^2^Département Formation Recherche (DFR) Santé, Université de Guyane, Cayenne, French Guiana; ^3^Service d’Anatomopathologie, Centre Hospitalier Andrée Rosemon, Cayenne, French Guiana; ^4^Equipe Opérationnelle d’hygiène hospitalière, Centre hospitalier Andree Rosemon Cayenne, Cayenne, French Guiana; ^5^Department of Dermatology, Centre hospitalier Andree Rosemon Cayenne, Cayenne, French Guiana; ^6^Service des Maladies Infectieuses et Tropicales, Centre hospitalier Andree Rosemon Cayenne, Cayenne, French Guiana; ^7^Laboratory, Centre hospitalier Andree Rosemon Cayenne, Cayenne, French Guiana; ^8^Unité Mixte de Recherche (UMR) Tropical Biome and Immunopathology, Université de Guyane, Cayenne, French Guiana; ^9^Service de Médecine, Centre hospitalier de l’Ouest Guyanais, Saint Laurent du Maroni, French Guiana; ^10^Département d’Information Médicale, Centre hospitalier de l’Ouest Guyanais, Saint Laurent du Maroni, French Guiana; ^11^Service d’endocrinologie diabétologie, Gastroentérologie, Centre Hospitalier Andrée Rosemon, Cayenne, French Guiana

**Keywords:** Histoplasmosis, Advanced HIV, adrenal gland, French Guiana, Immunosuppression, AIDS-related opportunistic infections

## Abstract

Adrenal histoplasmosis and primary adrenal insufficiency are mostly described in immunocompetent patients. This particular tropism is attributed to the presence of cortisol within the adrenal gland, a privileged niche for *Histoplasma* growth. In French Guiana, disseminated histoplasmosis is the main opportunistic infection in HIV patients. Our objective was to search in our HIV-histoplasmosis cohorts to determine how frequent adrenal insufficiency was among these patients. Between January 1, 1981 and October 1, 2014, a multicentric retrospective, observational study of histoplasmosis was conducted. Patients co-infected by HIV and histoplasmosis were enrolled in French Guiana’s histoplasmosis and HIV database. Among 349 cases of disseminated histoplasmosis between 1981 and 2014, only 3 had adrenal insufficiency (0.85%). Their respective CD4 counts were 10, 14 and 43 per mm3. All patients had regular electrolyte measurements and 234/349 (67%) had abdominal ultrasonography and 98/349 (28%) had abdominopelvic CT scans. None of these explorations reported adrenal enlargement. Overall, these numbers are far from the 10% reports among living patients and 80-90% among histoplasmosis autopsy series. This suggests 2 conflicting hypotheses: First, apart from acute adrenal failure with high potassium and low sodium, less advanced functional deficiencies, which require specific explorations, may have remained undiagnosed. The second hypothesis is that immunosuppression leads to different tissular responses that are less likely to incapacitate the adrenal function. Furthermore, given the general immunosuppression, the adrenal glands no longer represent a particular niche for *Histoplasma* proliferation.

## Introduction

Histoplasmosis was discovered over a century ago. Its severity varies with the intensity of exposure and host immunity. In immunocompetent individuals, presentations hence range from asymptomatic infections or mild pulmonary disease for low-intensity exposures, to severe pulmonary infections for heavy exposures. A chronic lung infection may develop with gradual loss of pulmonary function among patients with underlying lung disease, and if untreated, it will result in death. Among immunosuppressed patients the infection progressively disseminates to other organs causing non-specific syndromes. Various organs such as the lungs, gastrointestinal tract, bone marrow, central nervous system, liver or lymph nodes may thus be involved in the same patient. Histoplasmosis has been an AIDS defining infection since 1987. It is estimated to be one of the main opportunistic infections and cause of death among patients with advanced HIV in Latin America (Adenis et al., 2018). The literature reviews on histoplasmosis often mention adrenal histoplasmosis and primary adrenal insufficiency (Wheat, 1989; Larbcharoensub et al., 2011; Koene et al., 2013). Most cases are described in immunocompetent patients, and the explanation of this particular tropism is that the presence of cortisol within the *zona reticularis* and *zona fasciculata* of the adrenal gland constitutes a privileged niche for the unhampered growth of *Histoplasma*. Studies in immunocompromised patients with disseminated histoplasmosis suggest 10% of patients have adrenal involvement but, autopsy studies report that 80-90% of patients have adrenal involvement (Goodwin et al., 1980; Wheat, 1989). The adrenal gland is a richly vascularized organ that contains cortex and medulla. The cortex produces several steroid hormones and the medulla produces catecholamines. The early adrenal response to infection is an increase in the functioning of the hypothalamic-pituitary-adrenal axis leading to increased local and systemic corticosteroid levels and adrenocorticotrophin (ACTH). ACTH causes increased blood flow to the adrenal gland, which is a predisposing condition for hemorrhage (Salim et al., 1988). The hypercortisolism deregulates the normal immune response in the sites of inflammation, by altering the cytokine production and function, and decreasing the migration of effector cells. Moreover, the local production and release of glucocorticoids by the cortex and a relative lack of phagocytic reticuloendothelial cells facilitate the tropism of *H. capsulatum* for the adrenal gland. The destruction of the adrenal gland may later occur *via* the direct effect of *H. capsulatum* leading to vasculitis resulting in local ischemia and caseation necrosis (Roubsanthisuk et al., 2002). In advanced HIV disease, adrenal exhaustion, infection with opportunistic pathogens, and development of anti-corticosteroid and anti-adrenal gland cells antibodies (Salim et al., 1988; Sinha et al., 2011) often low level of ACTH are considered as potential mechanisms for progression to overt adrenal insufficiency even when no apparent lesion with medical imagery.

In French Guiana, disseminated histoplasmosis has been the main opportunistic infection and cause of death in HIV patients for decades. The impression of clinicians regarding adrenal failure is that it is seldom observed. Our objective was to search in our HIV-histoplasmosis cohorts to determine how frequent adrenal insufficiency was among these patients.

## The French Guiana HIV/Disseminated Histoplasmosis Cohort Experience

Between January 1, 1981 and October 1, 2014, a multicentric retrospective, observational study of histoplasmosis was conducted. Patients co-infected by HIV and histoplasmosis were enrolled in French Guiana’s histoplasmosis and HIV database. The inclusion criteria were: age >18 years, HIV infection; first proven episode of histoplasmosis following the EORTC/MSG criteria (De Pauw et al., 2008). Suspected but unproven histoplasmosis (patients cured by successful empirical antifungal therapy), diagnosis solely based on positive PCR, or histoplasmosis recurrence were not included. French Guiana is a French territory and as such hospitalized patients benefit from free explorations, treatments. Imagery such as ultrasonography, CT scanner, MRI are routinely prescribed for hospitalized patients in search of a diagnosis. Blood samples routinely measure electrolytes, notably sodium and potassium levels, which are markers of acute adrenal deficiency. The database was created in 1992. Incident HIV-associated histoplasmosis cases were included in the three hospitals of French Guiana. Sociodemographic, clinical, biological, immunovirological and therapeutic data were collected on a standardized paper form until October 2014: sex, age, place of birth, symptoms on admission, clinical entrance examination, immunovirological assessment, medical imaging, mycology, pathology, treatment received, duration, dosage, route of administration. Survival data was collected for the study period. STATA^©^ (College Station, Texas, USA) was used for the statistical analysis. The analysis was descriptive with frequencies and percentages for qualitative variables and median and interquartile range for quantitative variable.

The 1992 Histoplasmosis and HIV anonymized database has been approved by the French National Institute of Health and Medical Research institutional review board (CEEI INSERM) (IRB0000388, FWA00005831 18/05/2010), by the Comité Consultatif pour le Traitement de l’Information pour la Recherche en Santé(CCTIRS) (N° 10.175bis, 10/06/2010), and the Commission Nationale Informatique et Libertés (CNIL) (n ° JZU0048856X, 07/16/2010).

The median age was 39 years (interquartile range (IQR)=34-46), the median CD4 count was 31 per mm3 (IQR=12-70). Among 349 cases of disseminated histoplasmosis between January 1, 1981 and October 1, 2014, only 3 had adrenal insufficiency (0.85%). Their respective CD4 counts were 10, 14 and 43 per mm3. The first patient, a 30-year-old male from Suriname, died 34 days after the diagnosis of acute adrenal failure the reported cause of death was disseminated histoplasmosis. Among all patients with disseminated histoplasmosis all had regular electrolyte measurements and 234/349 (67%) had abdominal ultrasonography and 98/349 (28%) had abdominopelvic CT scans. CT scans became increasingly frequent over time (Chi2 for trend, P<0.001). Before 2006, 13/50(26%) were explored with a CT scan whereas after 2005 81/150 patients (53%) were explored with an abdominal-pelvic CT scan. None of these explorations reported adrenal enlargement.

## Discussion

Here in a hospital cohort of 349 cases of disseminated histoplasmosis in patients with advanced HIV disease the proportion of patients with adrenal deficiency was 0.85%, and among patients having benefitted from abdominal imaging, none had reports of adrenal enlargement. Overall, these numbers are far from the 10% reports among living patients and 80-90% among histoplasmosis autopsy series. Our retrospective analysis was a significant limitation, and all patients were not explored in the same manner depending on their clinical presentation (for example, cases with diarrhea benefitted from colonoscopy whereas those with enlarged superficial lymphadenopathies were more likely to have lymph node aspiration or biopsy) or the availability of different diagnostic tools at the time of their diagnosis, possible sources of information bias (Nacher et al., 2020). In French Guiana, fungal culture was first implemented in 1998, liposomal amphotericin B became available in 2003, and, perhaps more importantly for the capacity to detect adrenal enlargement, over time the use of thoraco-abdomino-pelvic CT-scan became increasingly frequent. These factors could therefore lead to a cohort effect but given the relative frequency of blood electrolyte measurements and decades of CT-scans, it seems likely that patent adrenal failure and adrenal enlargement are indeed rare.

Adrenal involvement is not necessarily associated with functional adrenal insufficiency. A literature review of 242 patients with adrenal histoplasmosis spanning 41 years found that 41.3% had adrenal hypofunction (Koene et al., 2013). It was often diagnosed incidentally. During active tuberculosis the adrenal glands are one of the 5 main organs involved (Lam and Lo, 2001). A large autopsy study in Hong Kong found 52/871 (5.9%) patients with adrenal involvement. Of the 52, 7(13.4%) had Addison’s disease due to bilateral involvement. Caseous necrosis and granulomatous inflammation with Langhan’s giant cells were seen in 71% and 40% of patients, respectively (Lam and Lo, 2001). Histoplasmosis lesions in immunocompetent hosts also leads to granulomatous inflammation and proliferation which explains the frequently enlarged adrenal glands.

Histopathological lesions reflect host reactions against *H. capsulatum*. They are classified into 4 categories: (i) tuberculoid, (ii) anergic, (iii) mixed and (iv), *sequelae*. Tuberculoid lesions usually correspond to a low inoculum and effective host tissular response. Anergic responses are observed in HIV patients and there is scarce or no tissue response. Local macrophages are inactive. Typically, there is an abundance of intracellular and extracellular yeast. The mixed form is intermediate between tuberculoid and anergic. One interpretation of our results is that given the intense immune suppression in our cohort the tissular response does not lead to adrenal enlargement, which would explain why imagery (ultrasonography or CT scan) remained negative. However, functionally, in hospitalized patients that can benefit from any exploration in a European hospital, in a hot tropical country, (Hahner et al., 2010; Hahner et al., 2015) conditions which would be predicted to precipitate acute adrenal failure, we only found 3 cases. This suggests 2 conflicting hypotheses: First, apart from acute adrenal failure with high potassium and low sodium, less advanced functional deficiencies, which require specific explorations may have remained undiagnosed. This suggests that systematic testing –instead of clinically oriented explorations— of the adrenal response would have unmasked more cases and that there may be a need for systematizing screening protocols. The second hypothesis, is that immunosuppression leads to different tissular responses that are less likely to incapacitate the adrenal gland function. Furthermore, given the general immunosuppression, the adrenal glands no longer represent a particular niche for *Histoplasma* proliferation. While the second hypothesis is hardly testable, the first one seems quite feasible and systematically testing the adrenal function would allow to determine whether the rarity of diagnoses of adrenal involvement in patients with advanced HIV is evidence of absence of adrenal insufficiency or whether the lack of systematic explorations is simply absence of evidence.

## Data Availability Statement

Upon approval by the Commission Nationale Informatique et Libertés, the raw data supporting the conclusions of this article will be made available by the authors, without undue reservation.

## Ethics Statement

The studies involving human participants were reviewed and approved by French National Institute of Health and Medical Research institutional review board (CEEI INSERM) (IRB0000388, FWA00005831 18/05/2010). The patients/participants provided their written informed consent to participate in this study.

## Author Contributions

MN-conception, first draft writing and final draft approval; KDA PC-validation and and editing; PA, AA, DB, RB, FD, MD, LE, CM, BN, NS, AV-review, investigation. All authors contributed to the article and approved the submitted version.

## Conflict of Interest

The authors declare that the research was conducted in the absence of any commercial or financial relationships that could be construed as a potential conflict of interest.
